# Pleomorphic adenoma gene 1 mediates the role of karyopherin alpha 2 and has prognostic significance in hepatocellular carcinoma

**DOI:** 10.1186/s13046-014-0061-1

**Published:** 2014-07-25

**Authors:** Zhe-yuan Hu, Sheng-xian Yuan, Yuan Yang, Wei-ping Zhou, Hua Jiang

**Affiliations:** 1 The Department of Plastic Surgery, Changzheng Hospital, Second Military Medical University, No.415 Fengyang Road, Shanghai, 200001, P.R. China; 2 The Third Department of Hepatic Surgery, Eastern Hepatobiliary Hospital, Second Military Medical University, Shanghai, China

**Keywords:** PLAG1, Nucleus shuttling, Tumor growth, Metastasis, Prognosis, Biomarker

## Abstract

**Background:**

Karyopherin alpha 2 (KPNA2) promotes tumor growth in hepatocellular carcinoma (HCC). We aimed to determine the content and clinical significance of mechanism underlying.

**Methods:**

The association of transcriptional factor pleomorphic adenoma gene 1 (PLAG1) with KPNA2 was explored by co-immunoprecipitation. *In vitro* gain- and loss-of-function models were established to explore the functional interaction. Clinical samples from 314 HCC patients were applied to explore the clinical significance.

**Results:**

We found that PLAG1 could associate with KPNA2 and be promoted into nucleus by KPNA2. The increment of proliferative and metastatic abilities by KPNA2 over-expression can be significantly retarded by PLAG1 inhibition. The co-enrichment of KPNA2 and PLAG1 in nucleus is observed in clinical samples and can distinguish patients with the worst prognosis. The positive PLAG1 expression is an independent risk factor of recurrence free survival (HR: 1.766, 1.315-2.371; P = 0.000) and overall survival (HR: 1.589, 1.138-2.220; P = 0.007). Especially for patients with positive KPNA2 staining (N = 152), the positive PLAG1 expression is the sole risk factor for both recurrence free survival (HR: 1.749, 1.146-2.670; P = 0.010) and overall survival (HR: 1.662, 1.007-2.744; P = 0.047).

**Conclusions:**

The nuclear import of PLAG1 by KPNA2 is essential for the role of KPNA2 in HCC cells and is significant to predict poor survival of HCC patients after hepatectomy.

## Background

Hepatocellular carcinoma (HCC) is the third leading cause of cancer deaths worldwide, with the incidence on the rise [1]. The survival of HCC patients after resection remains poor, mainly attributing to frequent metastases and recurrence [2]. Recently, plenty of researches have performed to explore mechanisms underlying the initiation, propagation and development of HCC [3],[4]. However, the complexity of HCC need further hypothesis-drove researches to be exerted. Dysfunction of the cellular transport machinery is commonly observed in multiple cancers including HCC [5]. Although some molecules are able to diffuse through the large Nucleus Pore Complexes (NPCs) in the nucleus membrane, factors larger than 45 kDa including that associated with malignant diseases need to be mediated by karyopherin to import into the nucleus [6]. Karyopherin alpha 2 (KPNA2) is one of karyopherin a family, and could form heterodimer with Karyopherin 1 to promote nucleus protein import as an adapter protein [7]. Recent studies have illustrated that KPNA2 might be a critical oncogene and a potential prognostic biomarker in malignant diseases including HCC [8]–[11]. Furthermore, KPNA2 knock-down could significantly inhibit HCC proliferation [12]. But till now, the mechanistic evidence of KPNA2 in HCC was obscure and deserved to be explored.

Transcriptional factors are widely involved in cancers and are bound to be enriched in nucleus. It raised the hypothesis that KPNA2 might affect cancer cells through the translocation of cancer-associated transcriptional factors. Previous report has indicated the direct association of KPNA2 with a zinc-finger transcription factors pleomorphic adenoma gene 1 (PLAG1) by the yeast two-hybrid system [13], suggesting PLAG1 might be one of critical mediators of KPNA2 effects in malignant diseases. PLAG1 was identified as a candidate oncogene in various malignant cancers. Recent report illustrated the over-expression of PLAG1 in hepatoblastoma, suggesting a potential role of PLAG1 in liver malignant disease [14]. Besides, insulin-like growth factor 2 (IGF-II), cellular retinoic acid binding protein (CRABP2) and cytokine receptor-like factor 1 (CRLF1), which are confirmed targets of PLAG1, might be involved in pathological process of HCC [15],[16]. However, whether KPNA2 might associate with PLAG1 and assist PLAG1 nucleus import to activate downstream effectors in HCC remains unclassified. Here, we explored the functional interaction of KPNA2 with PLAG1 and the clinical significance of the mechanism in HCC.

## Methods

### Clinical specimens and follow-up

The study protocol was approved by the clinical research ethics committee of Second Military Medical University (Shanghai, China). Written informed consent was obtained from all patients according to the policies of the committee. Information that could identify the patients was not included in this article.

The tissue microarray (TMA) were constructed as described previously [17]. Tumoral and corresponding non-tumoral tissues are separately deposed in different slices. A HCC cohort with 314 patients was used for immunohistochemical staining in TMA. All those HCC patients received curative hepatectomy at Eastern Hepatobiliary Surgery Hospital between July 5, 2003 and June 30, 2006. All HCC specimens were obtained immediately after hepatectomy and tissues were then fixed in 10% buffered formalin and embedded in paraffin.

The preoperative diagnosis and surgical procedure of HCC patients was carried out as described previously [18]. The clinical characteristics of HCC cohort are listed in Table. The differentiation of HCC was defined according to the criteria proposed by Edmondson and Steiner. Micro-metastases were defined as tumors adjacent to the border of the main tumor and were only observed under the microscope.

All prognostic information of HCC patients were checked by phone every 2-3 months during the first 2 years and every 3-6 months thereafter until follow-up ended on October 28, 2010. Two physicians who were unaware of the study performed follow-up examinations. Serum AFP levels and abdominal ultrasound examinations were performed for every month during the first year after surgery and every 3-6 months thereafter. A computed tomography and/or magnetic resonance imaging examination was performed every 3-6 months or when a recurrence were suspected. A diagnosis of recurrence was based on preoperative diagnosis criteria. Once recurrence was confirmed, further treatment was implemented depending on the tumor’s diameter, number, location, and vessel invasion as well as the liver function and performance status.

### Cell lines

The Huh7 and SMMC-7721 cells were cultured at 37°C in an atmosphere containing 5% CO2 in Dulbecco’s Modified Eagle’s Medium (DMEM) or Modified Eagle’s Medium (MEM) supplemented with 10% fetal bovine serum.

### Extraction of RNA, preparation of cDNA and quantitative real-time PCR (qRT-PCR)

Total RNA were extracted using Trizol reagent (Takara, Dalian, China) according to the manufacturer’s instructions. The quality of the total RNA was assessed by a Nanodrop 2000 and agarose gel electrophoresis. First-strand cDNA was synthesized from 1-2 μg of total RNA using random primers and the M-MLV Reverse Transcriptase (Invitrogen, CA).

Real-time PCR was performed using a StepOne Plus system (Applied Biosystems, Foster City, CA) with ACTB as endogenous control. The relative mRNA levels were calculated based on the Ct values and normalized using the ACTB expression. The sequences of primers are listed as: ACTB, Forward: AGTTGCGTTACACCCTTTCTTG, Reverse: GCTGTCACCTTCACCGTTCC; KPNA2, Forward: TGATGGTCCAAATGAACGAAT, Reverse: CTGGGAAAGACGGCGAGTG; CRLF1, Forward: TGGCTCTCTTTACGCCCTATTGA, Reverse: TGGCTTGAAAGAGGAAATCCTT; CRABP2, Forward: TGGGGGTGAATGTGATGCTG, Reverse: ACGGTGGTGGAGGTTTTGAT; IGF-II, Forward: AACTGGCCATCCGAAAATAGC, Reverse: TTTGCATGGATTTTGGTTTTCAT.

### Protein preparation and Western Blot analysis

Total proteins were extracted using RIPA Lysis Buffer and PMSF (Beyotime Co., China) according to the manufacturer’s instructions. Nucleus proteins were prepared using Cytoplasmic and Nucleus Protein Extraction Kit (Fermentas). Antibody dilutions were 1:2000 for KPNA2 (BD, USA), 1:200 for PLAG1 (Biossy, USA), 1:1000 for Lamin B (Santa Cruz) and 1:5000 for ACTB (Sigma-Aldrich, USA), respectively. Antibody binding was detected using an Odyssey infrared scanner (Li-Cor Biosciences Inc).

### Construction of in vitro gain or loss-of-function models

Expression vector encoding the human KPNA2 genes were purchased from Fulen Gen Company (Guangzhou, China). SiRNAs targeting to KPNA2 and PLAG1 were synthesized by GenePharma Company (Shanghai, China). The sequences of siRNAs were disclosed as: KPNA2-Si144: sense, 5’-ACGAAUUGGCAUGGUGGUGAATT-3’, and antisense, 5’-TTUGCUUAACCGUACCACCACUU-3’; KPNA2-Si467: sense, 5’-CCGGGUGUUGAUUCCGAATT-3’, and antisense, 5’-TTGGCCCACAACUAAGGCUU-3’; PLAG1-Si: sense, 5’-GCACAUGGCUACUCAUUCUTT-3’, and antisense, 5’-TTCGUGUACCGAUGAGUAAGA-3’.

KPNA2 expression vectors and siRNAs were transfected into HCC cells by Lipo2000 reagent (Life Technologies, USA) according to the manufacturer’s instructions. The expression of KPNA2 or PLAG1 in the transfected cells was examined by RT-PCR and Western Blot after 48 h. Cells transfected with empty vector or a scrambled siRNA were used as negative controls. We acquired cell clones with KPNA2 over-expression using puromycin.

### Cell proliferation assay

Approximately 2 × 10^3^ HCC cells were plated in 96-well plates. Cell proliferation was assessed using the Cell Counting Kit-8 (Dojindo Laboratories, Kumamoto, Japan) according to the manufacturer’s protocol. All of the experiments were performed in triplicate. The cell proliferation curves were plotted using the absorbance at each time point.

### Transwell assay

The 24-well Boyden chamber with 8-μm pore size polycarbonate membrane (Corning, NY) was used to analyze the migration of tumor cells. Approximately 1 × 10^4^ HCC cells were plated into chamber. HCC cells were plated into chamber 36 h after siRNA transfection (for both KPNA2 and PLAG1). About 24 hours later, the non-migrating cells on the upper chambers were removed using a cotton swab and migratory cells were stained. Cell number were plotted as the average number of migrated cells from 5 random microscopic fields.

### Co-immunoprecipitation (Co-IP)

Cell lysates were prepared from SMMC7721 and Huh7 cells without any KPNA2 manipulation. KPNA2 polyclonal antibody described above was diluted 1:1000. Co-immunoprecipitation was performed according to manufacture of Pierce Classic IP Kit (USA). Briefly, the protein extracts were incubated with either a specific primary antibody or a IgG control antibody overnight at 4°C. Five percent of whole cell lysates was saved as input controls. Immune complexes were collected on Protein A agarose. After washing three times with 0.7 ml of protein lysis buffer, the precipitates were boiled and analyzed using SDS/PAGE (10–12% gel) followed by western blotting to analyze the protein.

### Immunocytochemistry

Huh7 Cells with KPNA2 over-expression and negative controls (1:2) were plated into chamber. After 36 h, cells were fixed with 1% paraformaldehyde for 5 min at room temperature. For immunostaining, PLAG1 antibody (Aogma, USA), KPNA2 antibody (BD Biosciences), DAPI (Invitrogen, USA) and cross-adsorbed secondary antibodies were used. Fluorescence was detected using a Zeiss LSM 510.

### Immunohistochemical analysis

The immunohistochemical staining was performed on the TMA using a two-step immunoperoxidase technique. The KPNA2 polyclonal antibody (BD, USA) diluted 1:1000 and PLAG1 polyclonal antibody (Biossy, USA) diluted 1:200 were used as primary antibody. Briefly, after heating the sections in 10 mmol/L citrate buffer for antigen retrieval, sections were incubated first with primary antibodies, and then with secondary antibody for an hour at room temperature. The staining was assessed by two separate investigators who were blind to the patient characteristics. The positive KPNA2 and PLAG1 staining was defined as nucleus staining in more than 5% cells [12].

### Statistical analysis

We defined the recurrence-free survival (RFS) and overall survival (OS) as the interval of tumor resection to the detection of tumor recurrence and the subject’s death of HCC. All statistical analyses were carried out using SPSS version 16.0 software. A one-way analysis of variance, the chi-square test and the two-tailed Student’s t-test were performed when appropriate. Survival curves were calculated using the Kaplan-Meier method and compared using a log-rank test. P-value less than 0.05 were considered to be statistically significant.

## Results

### Transcriptional factor PLAG1 is promoted into nucleus by KPNA2

**Figure 1:**
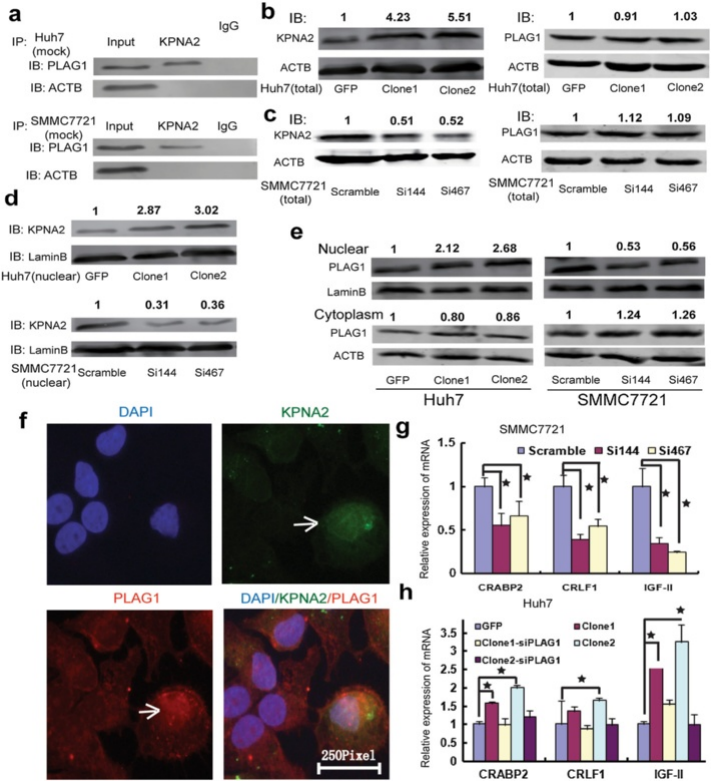
**Assistance of PLAG1 nucleus shuttling by KPNA2. (a)** The association of KPNA2 and PLAG1 was assayed by Co-IP, protein samples not proceeding to IP was designated as input and samples pulled down by IgG antibody was used as negative control. Unassociated protein ACTB was examined to exclude unspecific bind by KPNA2 antibody. **(b)** The expression of KPNA2 (left panel) and PLAG1 (right panel) total protein in control Huh7 cells (GFP) or Huh7 cells transfected with KPNA2 expression plasmids (Clone1 and Clone2). **(c)** The expression of KPNA2 (left panel) and PLAG1(right panel) total protein in control SMMC7721 cells (Scramble) or SMMC7721 cells transfected with KPNA2 siRNAs (Si144 and Si467). **(d)** Nucleus accumulation of KPNA2 could be manipulated by KPNA2 expression plasmids and siRNAs. **(e)** The nucleus accumulation (up panel) and cytoplasm expression (down panel) of PLAG1 in SMMC7721 and Huh7 cells. ACTB and Lamin B antibody were applied for endogenous antibody for total and nuclearnucleus protein determination respectively. **(f)** In situ observation of the nucleus accumulation of PLAG1 in Huh7 cell line was investigated by immunocytochemistry. Nucleus was stained by DAPI. Cells with KPNA2 overexpression was marked by the white arrows. **(g-h)** Expression of transcriptional targets of PLAG1 in SMMC7721 and Huh7 cells. Data represents as mean ± s.d. ★ represents statistical significance.

We applied co-immunoprecipitation using a polyclonal antibody of KPNA2 and proteins acquired from the assays were used for detection of PLAG1, with ACTB as a negative control. The association of PLAG1 with KPNA2 was confirmed in two HCC cell lines, as PLAG1, but not ACTB, could be detected in the precipitate enriched by KPNA2 antibody (Figure 1a). Next, *In vitro* models were applied to explore whether the association would be functional for PLAG1 in nucleus shuttling. Firstly, the overexpression of KPNA2 in Huh7 was validated in two different clones by stable transfection with KPNA2 expression vector (Figure 1b, designated as Clone1, Clone2). Then, we established a small-interfering RNA (siRNA)-mediated loss of KPNA2 expression in SMMC7721 cells (Figure 1c, designated as si144 and si467). KPNA2 acts as regulator of nucleus import, the translocation of KPNA2 into nucleus partly represented the biological effect of KPNA2 and was determined in HCC cell lines of in vitro models. Cell fractionation followed by immunoblotting indicated that intervention of KPNA2 could modulate the nucleus KPNA2 expression (Figure 1d), suggesting our in vitro models could be applied to investigate the role of KPNA2 in nucleus shuttling.

Nucleus and cytoplasm protein was extracted from HCC cell lines with KPNA2 manipulation and were applied for detection of PLAG1 protein. The results indicated that nucleus expression of PLAG1 could be significantly increased in Huh7 cells with KPNA2 overexpression. Besides, inhibition of KPNA2 could remarkably decrease the expression level of PLAG1 in nucleus (Figure 1e). Conversely, PLAG1 protein in cytoplasm was slightly decreased after ectopic over-expression of KPNA2 and was mildly increased by inhibition of KPNA2 (Figure 1e), which were consistent with the result that PLAG1 expression remained unchanged after manipulation of KPNA2 (Figure 1b-c). Immunocytochemistry was applied to observe the increased nucleus shuttling of PLAG1 in Huh7 cells with over-expressed KPNA2 compared with control Huh7 cells (Figure 1f). We then sought to validate the association between KPNA2 and PLAG1 by investigating the transcriptional regulation of downstream molecular by PLAG1. Several definite targets of PLAG1 were analyzed by qRT-PCR. Remarkably, we observed that the expression of IGF-II, CRABP2 and CRLF1 were significantly inhibited by KPNA2 siRNAs in SMMC7721 cells (Figure 1g). Increment of IGF-II, CRABP2 and CRLF1 were induced by KPNA2 over-expression in Huh7 cells (Figure 1h). Furthermore, we transfected PLAG1 siRNA into Huh7 cells of KPNA2 over-expressed clones and found that transcriptional up-regulation of IGF-II, CRABP2 and CRLF1 were significantly counteracted by PLAG1 inhibition (Figure 1h). In sum, we revealed that KPNA2 might act as a vehicle to transport PLAG1 into nucleus to regulate downstream effectors.

### PLAG1 mediate the role of KPNA2 in proliferation and migration of HCC

**Figure 2:**
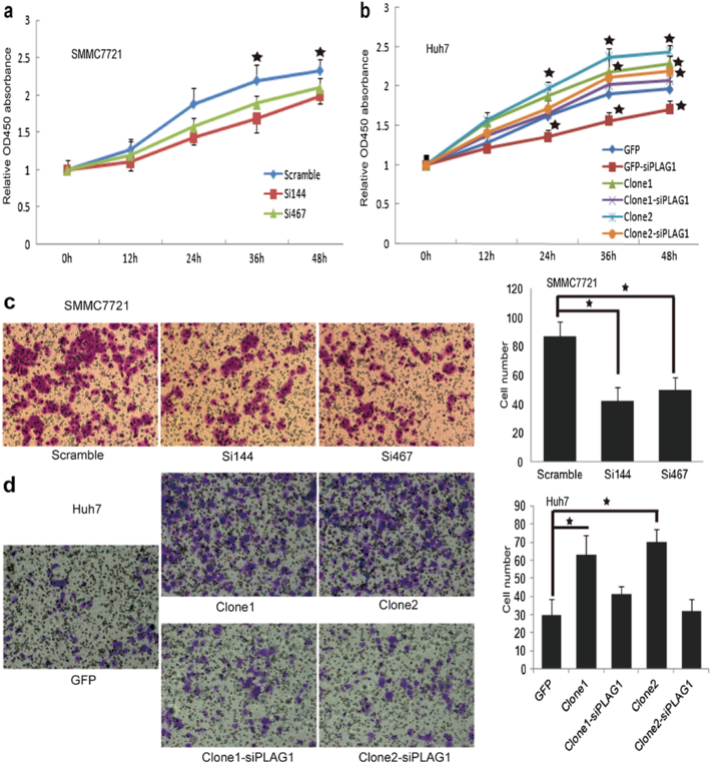
**PLAG1 is essential for the role of KPNA2 in proliferation and invasion of tumor cells. (a-b)** The cell proliferation of HCC cells was assayed every 12 hours for two days in three independent experiments. ★ represents statistical significance compared to Scramble or GFP cells. **(c-d)** The number of migratory HCC cells was calculated with crystal violet staining and representative fields were exhibited. Bar graphs in left panel show mean the average count of six random microscopic fields and the mean SEM. ★ represents statistical significance.

We then aimed to explore whether the interaction between KPNA2 and PLAG1 had functional significance in HCC. Firstly, we measured the proliferative capability of tumor cells by CCK-8 assays. The proliferation of HCC cells was significantly retarded by KPNA2 inhibition (Figure 2a) and accelerated by KPNA2 overexpression (Figure 2b). It is noteworthy that PLAG1 inhibition could significantly counterweighed the effect of KPNA2 overexpression in Huh7 cells (Figure 2b). Evidences have revealed the involvement of IGF-II in metastasis of HCC cells [19],[20]; we then sought to determine whether KPNA2 could promote the metastasis of HCC cells through PLAG1. Transwell assay was applied to find that inhibition of KPNA2 lead to decrease of migratory cells by nearly 40-50% in SMMC7721 cell lines (Figure 2c). KPNA2 over-expression could remarkably increase the migratory ability of Huh7 HCC cells *in vitro* and PLAG1 knock-down could significantly offset the effect of KPNA2 over-expression in HCC cell metastasis (Figure 2d). Collectively, the results indicated that the role of KPNA2 in proliferation and migration relied on PLAG1.

### The co-enrichment of nucleus PLAG1 and KPNA2 *in vivo*

**Figure 3:**
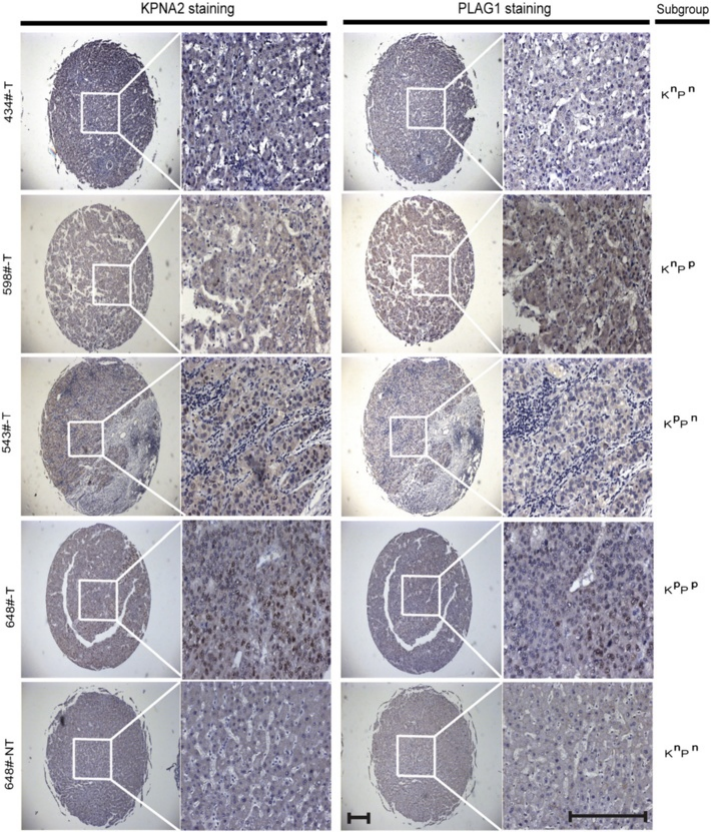
**The representative staining of KPNA2 and PLAG1 in clinical samples included in TMA.** IHC staining of four tumoral tissues (T) was shown to define four groups: K^n^P^n^, low KPNA2 and low PLAG1 enrichment in nucleus; K^n^P^p^, low KPNA2 and high PLAG1 enrichment in nucleus; K^p^P^n^, high KPNA2 and low PLAG1 enrichment in nucleus; K^p^P^p^, high KPNA2 and high PLAG1 enrichment in nucleus. One paired non-tumoral tissue (NT) was shown as control to tumoral tissues. Magnification scales represent 100 μm.

To determine the *in vivo* interaction and clinical significance of KPNA2 and PLAG1, we performed an immunohistochemical analysis of KPNA2 and PLAG1 in a tissue microarray including 314 HCC patients with tumoral (T) and corresponding non-tumoral (NT) in separate section (Table 1). Based on nucleus enrichment in cells of tumoral (T) and non-tumoral (NT) tissues, we defined the contents of KPNA2 and PLAG1 as positive or negative (Figure 3) and subdivided all patients into these groups: K^n^P^n^ (N_N_ = 117, N_NT_ = 235), negative KPNA2 and negative PLAG1 enrichment in nucleus; K^n^P^p^ (N_N_ = 45, N_NT_ = 68), negative KPNA2 and high PLAG1 enrichment in nucleus; K^p^P^n^ (N_N_ = 54, N_NT_ = 2) positive KPNA2 and negative PLAG1 enrichment in nucleus; K^p^P^p^ (N_N_ = 98, N_NT_ = 9), positive KPNA2 and positive PLAG1 enrichment in nucleus (Figure 3). Consistent with previous report [12], the positive KPNA2 expression was almost tumor specific, as only non-tumoral tissues of 11 HCC patients showed positive KPNA2 expression. Besides, the positive nucleus staining of PLAG1 in tumors was more frequent than in non-tumoral tissues (Table 2), further supporting the role of PLAG1 in HCC. Furthermore, we found that patients with positive KPNA2 expression were inclined to harbor positive PLAG1 expression in both T and NT tissues (Table 2). The results revealed the interaction between KPNA2 and PLAG1 *in vivo*.

**Table 1:** The clinico-pathological characteristics of patients according to nuclear enrichment of PLAG1

**Variate**	**PLAG1**^**▲**^		**P-value**
	**Negative**	**Positive**	
All cases	171	143	
Age (year), ≤60:>60	132:39	113:30	0.785
Gender, male:female	149:22	128:15	0.599
Child-Pugh, A:B	155:16	130:13	0.680
HBs antigen, positive:negative	150:21	123:20	0.737
HBe antigen positive:negative	35:136	31:112	0.889
AFP (ug/L), >20:≤20	62: 109	54: 89	0.815
Tumor size (cm), >5:≤5	81:90	88:55	0.030*
No. tumor, Solitary:Multiple	140:31	111:32	0.451
Edmondson Grade, I + II:III + IV	22:149	12:131	0.274
Vascular invasion, Present:Absent	99:72	88:55	0.564
Micro-metastases, Present:Absent	123:48	107:36	0.610

^▲^: PLAG1 status in tumoral tissues. *represents statistical significance.

**Table 2:** The co-enrichment of KPNA2 and PLAG1 in both tumoral (T) and non-tumoral (NT) tissues

**Staining**	**PLAG1**			**KPNA2**			**Correlation (PLAG1/KPNA2)**	
	**T**	**NT**	**P-value**^**▲**^	**T**	**NT**	**P-value**^**▲**^	**T**^**※**^	**NT**^**※**^
**Positive**	143	77	<0.001	152	11	<0.001	R=0.362	R=0.254
**Negative**	171	237		162	303		P-value <0.001	P-value <0.001

^▲^Represent the comparison of PLAG1 or KPNA2 nuclear staining between T and NT tissues.

^※^Represent the correlation of PLAG1 and KPNA2 nuclear staining in T or NT tissues.

### The tumoral PLAG1 expression correlates with survival of HCC patients

**Figure 4:**
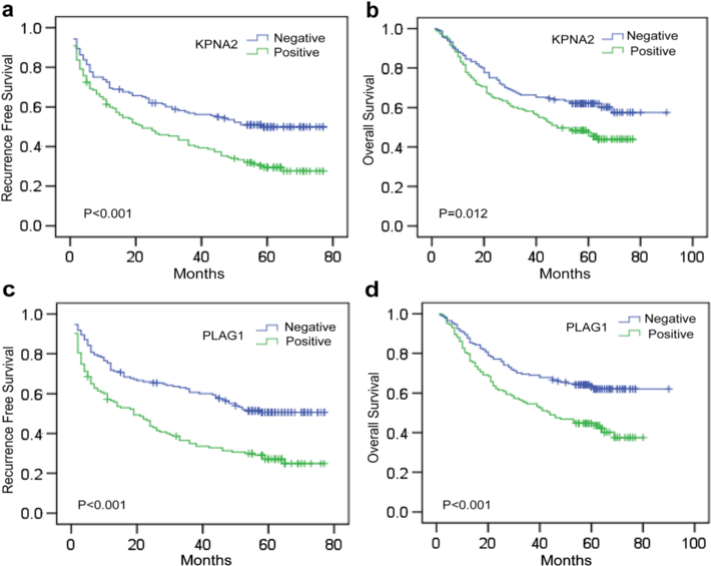
**The prognostic significance of KPNA2 and PLAG1 expression.** Kaplan-Meier analyses of recurrence-free survival **(a)** and overall survival **(b)** in HCC patients stratified by KPNA2 expression status. Kaplan-Meier analyses of recurrence-free survival **(c)** and overall survival **(d)** in HCC patients stratified by PLAG1 expression status. The survival curves were compared using a Long-rank test.

Previous report has indicated the clinical significance of positive KPNA2 in tumoral tissue as prognostic predictor. Consistently, we determined that HCC patients with positive KPNA2 expression in tumoral tissue would develop more frequent recurrence and death (Figure 4a-b). Given that PLAG1 is an indispensable mediator for the function of KPNA2 in HCC cells, we hypothesized that nucleus enrichment of PLAG1 in tumoral tissue might be a malignant character of HCC. Through analysis of the association between the PLAG1 expression and clinic-pathological characteristics, we determined that the positive PLAG1 expression was associated with larger tumor size (Table 1, P = 0.030). We then examined whether positive PLAG1 expression level correlated with outcome of HCC patients after hepatectomy. We found that patients with positive PLAG1 expression would have poorer prognosis including recurrence free survival (RFS, Figure 4c) and overall survival (OS, Figure 4d) of HCC patients after hepatectomy. Multivariate analysis revealed that PLAG1 could be an independent factor for RFS and OS of HCC patients after hepatectomy (Table 3). In sum, the result indicated that PLAG1 was a novel prognostic predictor for HCC patients.

**Table 3:** The clinico-pathological characteristics of patients with positive KPNA2 expression when grouped by nuclear enrichment of PLAG1

**Variate**	**PLAG1**^**▲**^		**P-value**
	**Negative**	**Positive**	
All cases	53	99	
Age (year), ≤60:>60	38:15	82:17	0.143
Gender, male:female	48:5	87:12	0.789
Child-Pugh, A:B	46:6	85:10	1.000
HBs antigen, positive:negative	47:6	86:13	0.803
HBe antigen positive:negative	7:46	22:77	0.201
AFP (ug/L), >20:≤20	20: 33	36: 63	0.862
Tumor size (cm), >5:≤5	30:23	67:32	0.005*
No. tumor, Solitary:Multiple	44:9	81:19	0.607
Edmondson Grade, I + II:III + IV	3:50	8:91	0.748
Vascular invasion, Present:Absent	35:18	67:32	0.858
Micro-metastases, Present:Absent	41:12	72:27	0.566

^▲^: PLAG1 status in tumoral tissues. *represents statistical significance.

### The positive PLAG1 expression is the only predictor for survival of KPNA2-positive HCC

**Figure 5:**
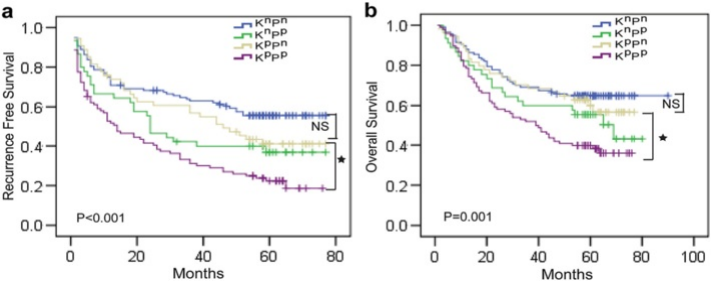
**The prognostic significance of the interaction between KPNA2 and PLAG1.** Kaplan-Meier analyses of recurrence free survival **(a)** and overall survival **(b)** of HCC patients divided into four subgroups described in Figure 3. The survival curves were compared using a Long-rank test. ★ represents statistical significance; NS represents no significance.

Furthermore, we found that patients with positive KPNA2 and positive PLAG1expression (K^p^P^p^) in tumor have the poorest RFS and OS compared to other groups (Figure 5a-b), suggesting the combination of high KPNA2 and PLAG1 density in nucleus would add accuracy to predict the prognosis of HCC patients. It is noteworthy that the differential prognosis between PLAG1-negative HCC patients with positive or negative KPNA2 staining shows no significance (Figure 5a, RFS: K^p^P^n^ vs K^n^P^n^, p = 0.226; Figure 5b, OS: K^p^P^n^ vs K^n^P^n^, p = 0.438), confirming the clinical importance of PLAG1 for the role of KPNA2 in HCC. However, for patients with positive KPNA2 expression, the status of PLAG1 in nucleus could significantly associate with tumor size (Table 3) and predict the RFS and OS (Figure 5a, RFS: K^p^P^n^ vs K^p^P^p^, p = 0.001; Figure 5b, OS: K^p^P^n^ vs K^p^P^p^, p = 0.001). Furthermore, multivariate analysis was applied to determine that the positive PLAG1 expression was the risk factor for prognosis of HCC patients (Table 4) and the only risk factor for prognosis of HCC patients with positive KPNA2 expression (Table 5). Collectively, the results revealed that PLAG1 was essential for clinical significance of KPNA2 and would add accuracy to stratify HCC patients with poor prognosis.

**Table 4:** Multivariate analyses of the recurrence-free survival (RFS) and overall survival (OS) in 314 HCC patients stratified by PLAG1 status

**Variate**^**▲**^	**RFS**		**OS**	
	**HR (95% CI)**	**P value**	**HR (95% CI)**	**P value**
Tumor size, >5 cm	1.061 (1.019-1.105)	0.004	1.081 (1.037-1.128)	0.000
Vascular invasion	1.379 (1.005-1.893)	0.046	1.386 (0.965-1.989)	0.077
HBe antigen positive	--	-	1.543 (1.068-2.229)	0.021
No. tumor: multiple	1.444 (1.108-1.880)	0.006	1.484 (1.141-1.930)	0.003
PLAG1 Positive	1.766 (1.315-2.371)	0.000	1.589 (1.138-2.220)	0.007
Edmondson Grade, III + IV	1.139 (0.652-1.987)	0.648	0.953 (0.507-1.791)	0.882

^**▲**^Variates significant in Univariate analyses were applied here.

*HR*, Hazard ratio; *CI*, Confidence interval.

**Table 5:** **Multivariate analyses of the recurrence-free survival (RFS) and overall survival (OS) in HCC patients with positive KPNA2 expression (K**^**P**^**P**^**n**^**VS K**^**p**^**P**^**p**^**, N = 152)**

**Variate**^**▲**^	**RFS**		**OS**	
	**HR (95% CI)**	**P value**	**HR (95% CI)**	**P value**
Tumor size, >5 cm	--	--	1.062 (0.757-1.121)	0.157
Vascular invasion	1.361 (0.898-2.064)	0.146	1.274 (0.785-2.067)	0.327
HBe antigen positive	1.267 (0.799-2.010)	0.315	1.387 (0.834-2.308)	0.208
No. tumor: multiple	1.227 (0.845-1.784)	0.282	1.183 (0.801-1.747)	0.399
PLAG1 Positive	1.749 (1.146-2.670)	0.010	1.662 (1.007-2.744)	0.047

^**▲**^Variates significant in Univariate analyses were applied here.

*HR*, Hazard ratio; *CI*, Confidence interval.

## Discussion

The nucleus transport system circulates various signaling molecules between the cytoplasm and nucleus. Karyopherins are one group of carrier proteins involved in the selective nucleocytoplasmic transport. Accumulating evidences have identified the critical roles of karyopherins in malignant diseases and KPNA2 gains the most attention [21]–[23]. Previous report has measured the gene expression profiling of karyopherins in HCC and found overexpressed KPNA2 could promote the proliferation of HCC cells [7]. Here, our results demonstrated that KPNA2 could significantly enhance the migratory ability of HCC cells. However, *in vivo* evidences should be acquired to support our results in the future.

One of the prominent of the cargo proteins of KPNA2 is the transcriptional factor PLAG1, previous evidence has illustrated that pleomorphic adenoma gene 1 (PLAG1) could be identified to be associated with KPNA2 *in vitro* and proved that a predicted nuclear localization sequence (NLS) composed of short stretches of basic amino acids was essential for physical interaction of PLAG1 with KPNA2 [13]. Also, researchers have illustrated that the activation of PLAG1 is considered to play important roles in the pathogenesis of various types of cancers [24],[25]. Recent report indicates that PLAG1 might be involved in regulatory gene work of hepatoblastoma, malignant liver tumor commonly occurred in childhood [26], suggesting a potential role of PLAG1 in malignant liver diseases. However, the involvement of PLAG1 in the role of KPNA2 in HCC remains elusive. Collectively, we aimed to explore the association between KPNA2 and PLAG1. We found that the nucleus import of PLAG1 was aided by KPNA2 and would amplify the transcriptional activities of PLAG1 in HCC. Several genes including IGF-II, CRABP2, CRLF1, CRIP2, which are transcriptional targets of PLAG1, could be up-regulated by enhanced KPNA2. IGF-II is frequently up-regulated in HCC and was enriched in the proliferation subclass of the molecular classification of HCC [27]. Besides, inhibition of IGF-II could impair the proliferation and invasive activities of HCC cells [20]. Furthermore, inhibition of PLAG1 in cell clones with stable KPNA2 over-expression could abolish the up-regulation of these genes and could counteract the pro-tumoral effects of KPNA2. The result implied that downstream molecular of PLAG1 such as IGF-II might be partly responsible for the role of KPNA2 in HCC. Although we revealed PLAG1 would be a critical mediator for KPNA2, it is noteworthy that whether other transcriptional factors carried into nucleus by KPNA2 might participate in HCC regulation need to be explored.

Cancer classification using biomarkers may effectively define the risk of recurrence, which allows for the use of appropriate treatments to acquire a better prognosis. The prognosis of patients with positive KPNA2 expression could be clustered by the status of PLAG1 nucleus enrichment, validating that the biological effects of KPNA2 relied on the interaction with PLAG1. Besides, for the subgroup of patients with negative PLAG1 expression, the prognostic value of KPNA2 came to be lost, further confirming that inhibition of PLAG1 could significantly retard the role of KPNA2 in tumor growth and metastasis *in vitro* as shown in Figure 2b and 2d. Combined with nucleus enrichment of PLAG1, the positive KPNA2 status would be more accurate to predict the prognosis of HCC patients after hepatectomy. Patients with co-existence of positive KPNA2 expression and positive PLAG1 expression should be closely monitored and receive appropriate adjuvant therapies. However, further investigation should be done to validate the prognostic value of KPNA2 and PLAG1 in other cohort of HCC patients, which would be promising for clinical application to reduce the false positive rate to identify and monitor patients with high recurrent risk after hepatectomy.

## Conclusions

PLAG1 could be impelled into nucleus by interaction with KPNA2, adapter acting in nucleus protein import. Co-enrichment of KPNA2 and PLAG1 in nucleus is observed in clinical samples. The increment of proliferative and metastatic abilities by KPNA2 can be significantly retarded by PLAG1 inhibition. Positive expression of both PLAG1 and KPNA2 could predict prognosis of HCC patients after hepatectomy Furthermore, the positive PLAG1 expression is the only risk factor for recurrence free survival and overall survival by multivariate analyses of patients with positive KPNA2 expression, further indicating the clinical significance of PLAG1 interaction with KPNA2 and harbor great applicability to distinguish HCC patient to be closely monitored after hepatectomy.

## Competing interests

The authors declare that they have no competing interests.

## Authors’ contributions

ZYH, SXY, WPZ and HJ designed and supervised the experiments. ZYH, SXY and YY performed qRT-PCR, cell proliferation assay, Transwell assay and immunohistochemistry. YY and WPZ collected clinical samples and supervised clinic-pathological data. ZYH, SXY, WPZ and HJ performed statistical analysis and draft the paper. All authors have read and approved the final manuscript.
